# Kill-painting of hypoxic tumours in charged particle therapy

**DOI:** 10.1038/srep17016

**Published:** 2015-11-24

**Authors:** Walter Tinganelli, Marco Durante, Ryoichi Hirayama, Michael Krämer, Andreas Maier, Wilma Kraft-Weyrather, Yoshiya Furusawa, Thomas Friedrich, Emanuele Scifoni

**Affiliations:** 1 Biophysics Department, GSI Helmholtzzentrum für Schwerionenforschung, 64291 Darmstadt, Germany; 2 Research Center for Charged Particle Therapy and International Open Laboratory, National Institute of Radiological Sciences, 263-8555 Chiba, Japan; 3 Technical University Darmstadt, 64283 Darmstadt, Germany

## Abstract

Solid tumours often present regions with severe oxygen deprivation (hypoxia), which are resistant to both chemotherapy and radiotherapy. Increased radiosensitivity as a function of the oxygen concentration is well described for X-rays. It has also been demonstrated that radioresistance in anoxia is reduced using high-LET radiation rather than conventional X-rays. However, the dependence of the oxygen enhancement ratio (OER) on radiation quality in the regions of intermediate oxygen concentrations, those normally found in tumours, had never been measured and biophysical models were based on extrapolations. Here we present a complete survival dataset of mammalian cells exposed to different ions in oxygen concentration ranging from normoxia (21%) to anoxia (0%). The data were used to generate a model of the dependence of the OER on oxygen concentration and particle energy. The model was implemented in the ion beam treatment planning system to prescribe uniform cell killing across volumes with heterogeneous radiosensitivity. The adaptive treatment plans have been validated in two different accelerator facilities, using a biological phantom where cells can be irradiated simultaneously at three different oxygen concentrations. We thus realized a hypoxia-adapted treatment plan, which will be used for painting by voxel of hypoxic tumours visualized by functional imaging.

Hypoxia is a feature of most tumours, albeit with variable incidence and severity within a given patient population^1^. It is a negative prognostic and predictive factor owing to its multiple contributions to resistance to cell death (chemoresistance, radioresistance), angiogenesis, vasculogenesis, invasiveness, metastasis, altered metabolism and genomic instability^2^.

Hypoxia-induced radiation resistance is generally acknowledged as a major limiting factor for tumour control in radiotherapy^3^. The increase of radioresistance is quantified by the oxygen enhancement ratio (OER), i.e., the ratio of iso-effective doses in hypoxic and fully oxygenated conditions:




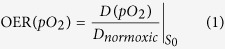




where *pO*_*2*_ defines a given level of hypoxia, i.e. the oxygen concentration (usually expressed as a partial pressure in %), *S*_0_ is a given cell survival fraction and *D* are corresponding radiation doses (usually expressed in Gy). Several *in vitro* studies demonstrated that for most of the cell lines irradiated with X-rays in anoxia (*pO*_*2*_ = 0) the OER is approximately 3.

Charged particle therapy is a cutting-edge radiotherapy technique which, among several physical and radiobiological peculiar advantages^4^,^5^, has the potential of a reduced OER. This is due to the special features of this type of radiation, characterized by a high linear energy transfer (LET, usually expressed in keV/μm), i.e. by dense ionization tracks, opposite to sparsely ionizing photons commonly used in radiotherapy^6^. The resulting direct DNA damage produces a high relative biological effectiveness (RBE) compared to photons, and is less sensitive to the presence of molecular oxygen. It was indeed demonstrated that OER decreases with LET, dropping to almost 1 over a few hundreds of keV/μm^7^,^8^. The OER reduction is generally attributed to recombination of the radiation-induced free radicals or production of “oxygen in the track”^9^,^10^,^11^,^12^. Particle therapy is therefore assumed to be especially effective against hypoxic tumours^13^, and can specifically target cancer stem cell subpopulations in hypoxic niches within the tumour^14^. A clinical study on radiotherapy of hypoxic uterine cervix cancers was performed using passively modulated carbon ions in Japan^15^. The results support the hypothesis that hypoxia is less important in radiotherapy using heavy ions compared to conventional photons, although the need for more research was pointed out in order to improve local control.

OER as a function of LET has been extensively measured, but only for fully anoxic conditions or for uncontrolled *pO*_*2*_^7^,^8^. However, tumours and normal tissues have oxygen concentrations in between 21% and 0%, a condition known as physioxia: 3%–7.4% in normal tissues, 0.3%–4% in tumours^16^. The regions at intermediate hypoxia are those more important in determining tumour response to radiotherapy^17^. Moreover, the normal LET distribution in a typical carbon ion irradiation is exceeding 100 keV/μm in a very small region of the target only. For this reason it is important to optimize the treatment planning system (TPS) accounting for both LET and *pO*_*2*_. This is possible for particle therapy using the active scanning dose delivery technique^18^. However, up to now, particle therapy TPS was always limited to optimization of the RBE-weighted dose^19^, without including any hypoxic effect, and especially any intra-tumour heterogeneous sensitivity.

The dependence on OER from *pO*_*2*_ was measured already many years ago for X-rays^20^ and can be described by the Alper and Howard-Flanders formula, derived from modeling a Michaelis-Menten kinetics mechanism




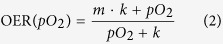




where *m* is a maximum effect and *k* a concentration corresponding to a half-maximum sensitization, i.e. the *pO*_*2*_ value corresponding to the flex point of the sigmoid-shaped curve.

Modern functional imaging provides quantitative information on hypoxia *in vivo*^21^,^22^, allowing intra-tumour heterogeneous mapping, especially through PET biomarker tracers, e.g. with ^18^F labelled nitroimidazoles such as misonidazole (^18^F-MISO), azomycin-arabinoside (^18^F-AZA) and the nucleoside analog (^18^F-HX_4_)^23^. Other non-invasive methods are recently emerging and include functional magnetic resonance imaging^24^, fluorescent markers^25^,^26^, Cherenkov radiation^27^. Despite the large amount of preclinical research in this field, a real use of hypoxia information in clinics is still missing^28^. Clinical implementation needs adaptive TPS^29^, identification of tumour sub-targets and methods for their selective irradiation. Intra-tumour heterogeneities can be targeted with a non-uniform dose prescription in the TPS, based on sub-volume boosting or *dose-painting* by numbers^30^. The reduced OER at high LET can be exploited in charged particle therapy using either protons^31^ or heavy ions^32^ with TPS where, by prescription, the high-LET components of the beam are constrained in the hypoxic regions: the so-called *LET-painting*. To integrate the information in the TPS, it is necessary to attain a full description of OER as a function of LET and *pO*_*2*_. A few models have been previously reported^33^,^34^,^35^,^36^, but the lack of experimental data did not allow their complete verification.

Here we develop an ion TPS optimized on the cell killing of the entire heterogeneous tumour volume (*kill-painting*), considering the intermediate levels of oxygenation and taking fully into account the biological advantages of ion beams, as well as the flexibility of the active scanning dose delivery system. We present the first set of systematic *in vitro* measurements of OER at intermediate values of oxygen concentration and LET. We then use the experimental data for validating a model describing the OER dependence versus *pO*_*2*_ and LET, which we implement in the ion TPS. Finally, we verify our new TPS approach predictions with measurements on extended heterogeneous targets including mammalian cells at different oxygen concentrations.

## Results

### Survival data collection

We performed clonogenic survival rate measurements on CHO cells at 5 different oxygen concentrations using photons and different ions at different dose-averaged LET 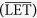, with a special focus in the region of maximum gradient of the OER (70–150 keV/μm). The measurements were done at the HIMAC accelerator of the National Institute for Radiological Sciences (NIRS) in Japan and were integrated with previous measurements performed in fully anoxic condition at the SIS18 of the GSI Helmholtz Center and the Heidelberg Ion Therapy (HIT) synchrotrons in Germany^37^, using 2 different hypoxic chambers and ions of different mass and velocity.

**Figure 1:**
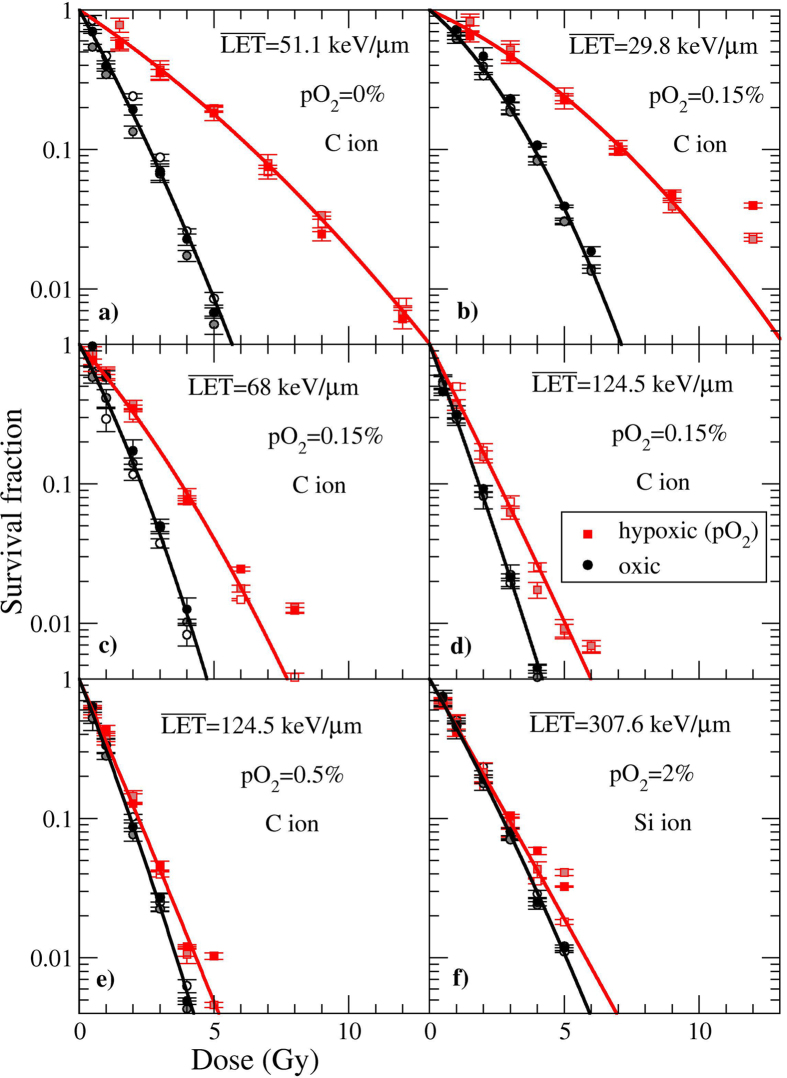
Oxic and hypoxic survival rates at different LET. A subset of the measured survival curves used to extract OER points, for different LET and *pO*_*2*_ values, including the corresponding (contemporary) oxic measurement. Each curve is obtained by fitting three independent measurements (different symbol colors), except the hypoxic curve in panel d, where only two measurements were considered.

Figure 1 shows examples of the measured survival data. The different filling of the points represents the three independent sets of measurements, while error bars are standard deviations obtained by averaging the three technical replicates. Each survival curve was fitted by the usual linear-quadratic (LQ) equation




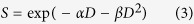




with fitting parameters *α* and *β*, by pooling all the biological replicates in a single set, thus accounting for the biological variability and obtaining a single set of fitting parameters (see Supplementary Information (SI) for details).

The OER values (eq. 1) were then calculated using the formula




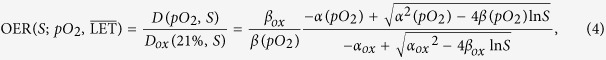




where the index *ox* defines parameters extracted from the corresponding normoxic survival curve (performed on the same day, see SI). The full dataset is shown in Table S1 together with all the information related to the experimental conditions and the corresponding OER(10%) values.

### OER model description and verification

We extended the Alper and Howard-Flanders (eq. 2) model^20^ by including the LET dependence. The LET variable is initially introduced in fully anoxic conditions according to an empirical formula developed in analogy to the classical formalism for *pO*_*2*_ dependence, where an additional exponential parameter (*γ*) takes into account the steeper dependence of the OER on the LET variable. This approach for the LET dependence is empirical, but the use of this formalism can be mechanistically justified within the *oxygen-in-the-track* theory, where the enhancement of sensitivity for rising LET is attributed to the nanoscopic production of molecular oxygen in highly dense tracks^38^, which is supralinear with respect to LET.

With this approach, the LET dependence in anoxic condition can be written as




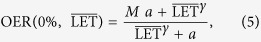




where *M*, the maximum possible effect for enhanced radioresistance, corresponds to an asymptotic level of low LET (X-rays) and oxygen concentration. The parameters *a* and γ are fitting parameters; *a*^1/ *γ*^ defines the LET value corresponding to half maximum of the sensitization (the flex point of the LET profile). We assume *a* and *γ* as tissue-independent parameters related to the radiation quality only. This is similar to what is observed for the RBE, whose absolute values are strongly tissue-dependent, but the position of the maximum on the LET scale is almost constant^39^.

For fitting these parameters, we initially used the extensive dataset measured at NIRS for V79 cells at *pO*_*2*_ = 0%^8^ and subsequently adapted it to the specific cell line subject of this study (CHO), leading to *a* = 8.27∙10^5^ (keV/μm)^γ^ and γ = 3.0. The maximum OER *M* was set to 2.7, based on our recent X-ray measurements on CHO cells^40^. This slightly lower value presented by these cells may be due to their specific characteristics in terms, e.g., of repair capabilities and scavengers´concentration.

**Figure 2:**
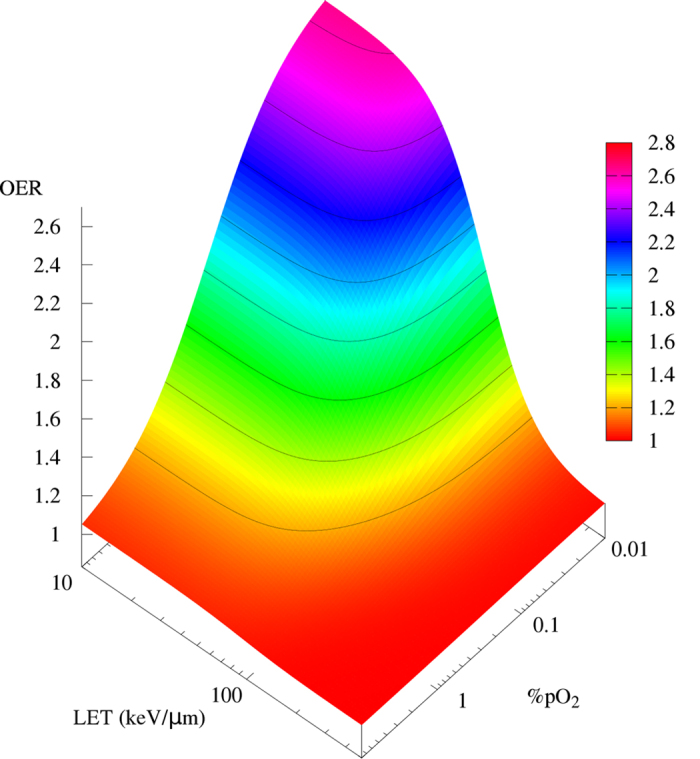
OER model. Two-dimensional semiempirical description of the OER dependence on LET and *pO*_*2*_ proposed in this work.

The oxygen concentration dependence can then be introduced into eq. 5 using the Alper formula in eq. 2 (Fig. 2):




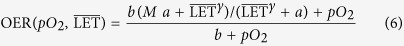




where the parameter *b* *=* *k*, is set to 0.25% according to a recent X-ray data parameterization^41^.

**Figure 3:**
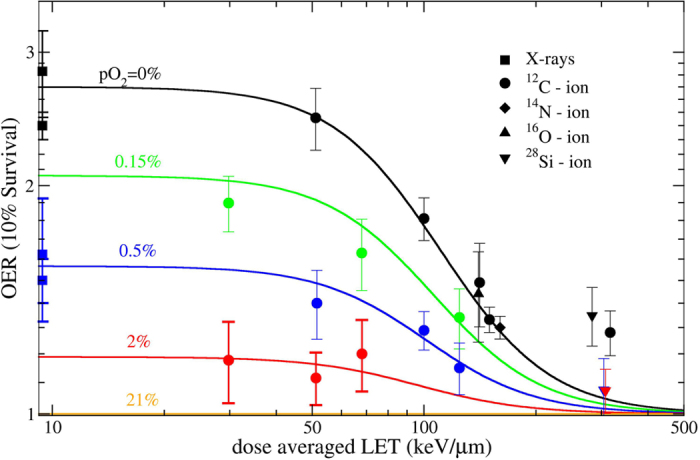
OER model verification. Collection of all OER measurements compared to model surface cuts.

Equation 6 describes the experimental data (Fig. 3) very well without any additional fitting parameter. The agreement is particularly good in the region of intermediate LET (50–150 keV/μm), where the surface shows its maximum gradient (Fig. 2), and which is more relevant for clinical applications. The few deviating points at very high LET are occurring in a region where probably the track-segment conditions are not fulfilled, or a high contamination of nuclear fragments is reducing the actual LET of the beam.

### The kill-painting approach

The OER model (Fig. 2, eq. 6) is then used for implementing an adaptive treatment planning for hypoxic tumours in TRiP98^42^,^43^, the research TPS on which most commercial systems currently used for C-ion therapy in several facilities in Europe and China are based. Since the dose dependence of OER is small^41^, especially for high LET^33^, the average value 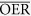, similar to OER(*S* = 0.1), can be used as a dose modifying factor at any survival level and properly introduced in the TPS.

While the mathematical details of the OER driven optimization in TRiP98 are available in the M&M section, we want to stress here that the optimization quantity is not anymore an RBE-weighted dose, but rather directly the surviving fraction *S*. The method is therefore distinct from dose- or LET-painting, where plan constraints (a given LET or dose) are prescribed by construction, e.g. by applying dose ramps^32^ and we chose to call it a “kill-painting”^44^.

**Figure 4:**
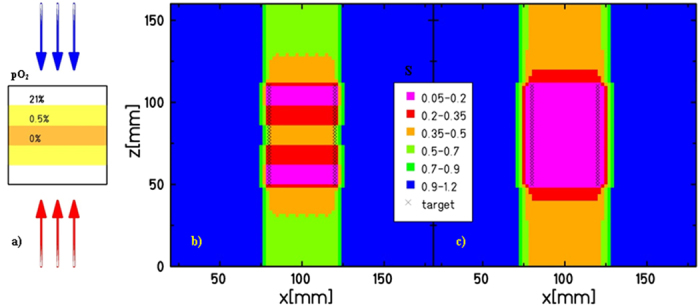
Kill-painting. Planned survival rates (values in legend) on the differently oxygenated target shown on panel (**a**), without (**b**) and with (**c**) considering the inhomogeneous oxygen concentration in the optimization.

An example is shown in Fig. 4, where the survival profile of a heterogeneous phantom, having a cellular target at 3 different levels of oxygenation (as illustrated in panel 4a) is plotted for a forward plan (panel 4b), optimized disregarding the oxygen effect, and for an inverse plan (panel 4c), where the oxygen concentration is specifically included in the optimization. In the panel 4b, the highly inhomogeneous survival is related to the size of the target, which “dilutes” the LET distribution. In a conventional plan, lower LET values are in the target center, where we placed the maximum hypoxia level, as it indeed generally occurs in real tumours. In the panel 4c, the prescribed survival level is restored and uniformly distributed all over the target, at a price of a slight increase of the dose in the entrance channel (corresponding to about 20%).

### Extended target irradiation

**Figure 5:**
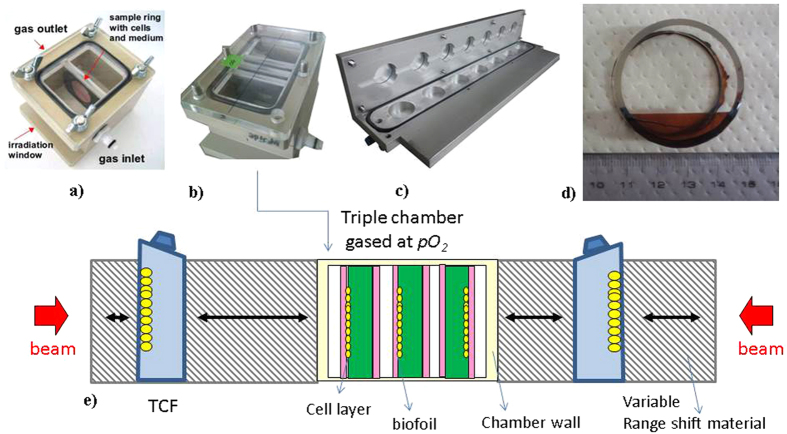
Experimental verification setup. Single (**a**) and triple (**b**) hypoxic chambers used for experiments in Germany (GSI and HIT); chamber set used at NIRS for survival curve experiments (**c**), holding several specific Petri dishes (**d**); “hypoxic phantom” (**e**) used for all the extended target measurements. The GSI triple chambers (**b**) were used both in Japan and Germany, and additional measurement points in normoxic regions were collected with normal tissue culture flasks (TCF).

We tested the new feature of our TPS with dedicated experiments on extended biological phantoms, both for its predictive capabilities (forward plans) as well as for its adaptive features (inverse planning), in recovering a uniform survival level. Figure 5e shows the experimental setup, where cellular samples on their turn at different oxygen concentrations can be irradiated with two opposite beams (see M&M for details).

#### a) Forward planning (Expected Survival)

**Figure 6:**
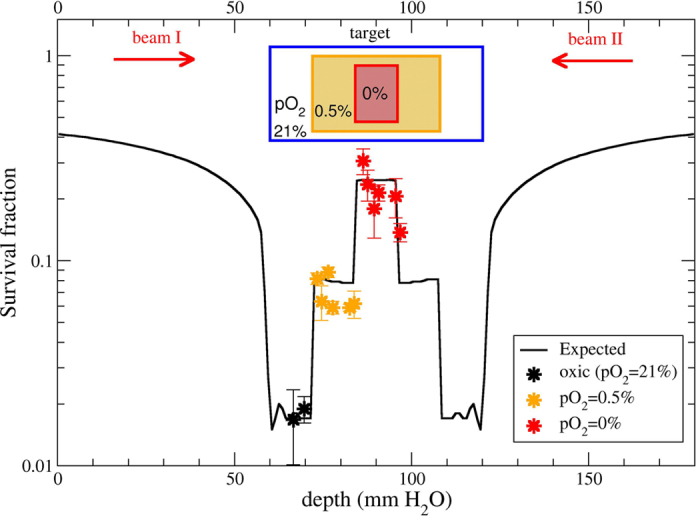
Non-optimized plan verification Extended target survival measurements performed at HIMAC compared with the calculation by our new TPS version. A beam of 290 MeV/u ^12^C-ions was used from both sides, passively modulated in a SOBP of 6 cm, on a phantom of 18 cm. Dose in the target was recalculated from the oxic control as 9.5 Gy (RBE) (see M&M).

In Fig. 6 we show the verification of the non-optimized plan. The forward planning test was performed at the HIMAC accelerator at NIRS with superimposition of two opposite passively-modulated carbon beams. The expected steep change in survival, from approximately 2% in normoxia up to almost 30% in anoxia, is reproduced by the measurements in the different regions of the phantom.

#### b) Inverse planning (OER- Optimization)

**Figure 7:**
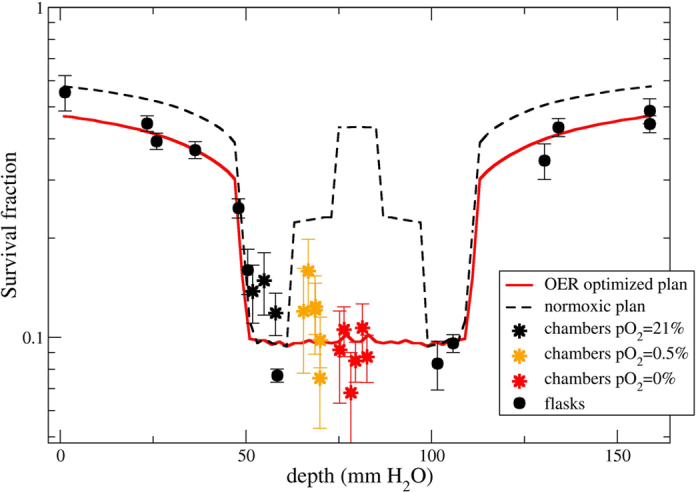
OER optimized plan verification. Comparison of expected survival in an OER optimized plan with experimental results, performed at GSI. An actively scanned ^12^C ion beam, composed of 17 monoenergetic slices ranging from 234.64 to 155.26 MeV/u was used from both sides. The target length was 6 cm on a phantom of 16 cm. The beam was optimized with a prescribed survival level in the target of 0.1, corresponding to a RBE-weighted dose of 6.5 Gy (RBE) in normoxia. RBE-weighted dose in the entrance is 2.8 Gy(RBE). The dashed curve represents the expected survival across the phantom, when a normoxic plan is applied (similar to previous figure). Absolute measured and calculated data are shown, with no recalculation adjustment applied.

In Fig. 7 we show the verification of the optimized plan performed at the SIS18 accelerator at GSI using carbon ions delivered by raster scanning and active energy modulation. The dashed line represents the corresponding survival prediction for an irradiation with a plan optimized disregarding the different oxygenation conditions, as in Fig. 6. The full red line is instead the result of the new calculation taking into account the intra-target heterogeneity in radiosensitivity, using kill-painting. The measured survival is approximately constant all over the target at different oxygen concentrations. The plan is correctly predicting the flat survival in the target region and the slight increased cell death in the beam entrance channel due to the increased dose.

We performed several additional experiments (see SI) for obtaining proper normoxic controls on the whole bio-phantom used. We irradiated normoxic cells, placed all along the target volume with a normoxic plan (Fig. S2a), to check the response of the phantom under the two fields, without any oxygen effect. Then we irradiated with an OER optimized plan normoxic cells placed in the same position where the anoxic ones were situated, i.e. at the center of the target (Fig. S2b), to verify the pure effect of the RBE-weighted dose of the modified plan. Furthermore, we compared the radiosensitivities of the different cell systems used (Fig. S3).

We finally analysed and compared the contributions of the two fields in the two different plans (see Fig. S4), showing how the optimization redistributes the particle fluences in the different energy slices. One of the effects of this redistribution is that the 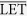 in the center of the target increases from 55 to 75 keV/μm. This shows that both dose and LET variations are contributing to the increased effectiveness achieved by the kill-painting.

### Therapeutic improvement assessment

The kill-painting approach described here is able to optimize a plan in particle therapy for a heterogeneous tumour target, i.e. with variable radiosensitivity. This is done by correctly predicting the heterogeneous response, voxel by voxel, and adapting the irradiation fields to restore a uniform survival, at the price of a slight increase of dose in the entrance channels, which is kept minimal by the optimization.

**Figure 8:**
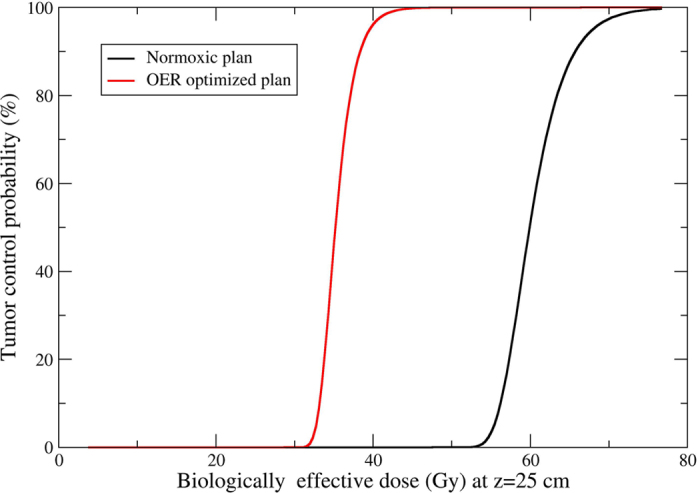
Kill-painting therapeutic improvement. Tumour control probability computed as a function of a biologically isoeffective dose (BED) for the two different plans. The BED is computed in the entrance channel at 25 mm depth, in order to indicate a similar damage to the normal tissue, after application of several fractions of each plan to the target.

A quantitative estimation of the overall effect of the OER-optimized plan, considering also the increased dose in the entrance channel, is shown in Fig. 8. The tumour control probability (TCP) for the idealized heterogeneous target phantom used in our experiments (Fig. 5e), assuming a radiotherapy course in *n* fractions is:




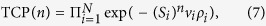




where *S*_*i*_ are the survival levels in each voxel *i* of the tumour after each dose fraction*, N* is the total number of voxels in the tumour*, v*_i_ are the voxel volumes (2 × 2 × 2 mm^3^ in this case, considering the pseudo-CT calculation grid used) and *ρ*_*i*_ the corresponding local number of cells per unit volume.

For comparison with the normoxic plan, we consider irradiation schedules giving the same biologically effective dose (BED) in the entrance channel. The BED is a conventional isoeffective quantity based on the LQ eq. 3, and can be computed by the Fowler formula^45^




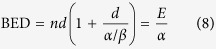




where *E* *=* −log *S(n), d* is the dose per fraction, *S(n)* the overall survival after *n* fractions and *α, β* are the parameters of a reference survival curve for the specific tissue (eq. 3). For equal fractions *E* *=* −*n* log *(S)*, thus a different number of fractions gives the same BED at a given depth in the normal tissue for the normoxic and adapted plan. The isoeffective number of fractions *n*’ for the normoxic plan, giving the same BED of *n* fractions of the OER-optimized plan in the voxel *k* can be computed as




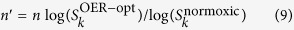




where *S*_*k*_ is the survival level, for each plan, in a given voxel *k* of the entrance channel and *n*’ < *n*.

In Fig. 8 we show the TCP (eq. 7) of the two plans as a function of the BED, where the BED is sampled at an average depth *z* = 25 mm, considering a constant *ρ*_*i*_ = 10^4^ cells/mm^3^. The ratio of the BED providing the same 50% TCP for the two plans is 1.78. We therefore expect a significant improvement in local control using an OER-optimized plan for hypoxic tumours also *in vivo*, at the same risk of normal tissue complications (BED in the entrance channel).

## Discussion

### Potential extensions and limitations

The adaptive TPS optimizes the use of charged particles for tumours with intermediate hypoxic levels. For ^12^C-ions, the LET values in the target region are not high enough to induce a complete re-sensitization (OER∼1) for totally anoxic cells, and this limitation of light ions is considered the reason of the limited improved local control in clinical trials^15^. Kill-painting provides an optimal redistribution of the LET components, according to the measured oxygen concentration, potentially leading to a much higher benefit. The dynamic nature of tumour hypoxia is of course challenging for an adaptive treatment plan. Our method allows fast recalculation of an optimized plan according to the changes of the tumour oxygenation in time. Reoxygenation, which has been indicated as a possible relevant issue^46^, can be included in the model, pending quantitative data from functional imaging. However, hypoxia is a major problem especially in hypofractionation schemes, where re-oxygenation is limited or completely missing for single-fraction high-dose treatments. Charged particles are ideal tools for hypofractionation (5), and indeed most clinical trials at NIRS use a very limited number of fractions of C-ions, down to single fractions for non-small cell lung cancers^13^. The adaptive TPS developed here is therefore a powerful tool for hypofractionated particle therapy.

The major limitations of the present approach, which relies on precise information on intra-tumour *pO*_*2*_ distribution, are related to the accuracy and reproducibility of the functional imaging techniques. One major problem which is in the course of investigation is the translation of PET uptake information to oxygen concentration data^47^,^48^.

The present OER model does not explicitly include a dependence on particle type, even if the TPS implementation supports this feature. Our measurements demonstrate that for light to intermediate weight ions (C, N, O), the track structure impact on OER is negligible, while it may be significant for heavier particles, such as Si (see triangles in Fig. 3). Kill-painting of hypoxic tumours will be more effective using heavier ions, such as oxygen^33^. Oxygen beams reach LET values over 200 keV/μm in the tumour, and having a mass very close to carbon, the same OER model we introduced here can be safely used. Since the high LET of oxygen beams may present counter-indications for the normal tissue, their use could be more advantageous in a mixed beam treatment, in combination with lower LET ions. In this connection, we recently enhanced our TPS with the possibility to combine different ions^44^ in a multi-ion optimization. Merging OER-driven and multi-ion optimization will extend the kill-painting approach with more degrees of freedom, providing useful protocols for specific clinical cases.

It is also important to stress that the implemented model for the OER dependence is semi-empirical, as we mentioned in the previous section, where the *γ* exponent of eq. 5 is here a fitting parameter. However its value (>1) seems to support the hypothesis related to the oxygen in the track theory that an increase of LET acts more than linearly as an increase of pO_2_. For example, in ref. 38, the local production of O_2_ in high LET track is obtained by including in the track structure calculation the double ionization processes. It is found that the O_2_ yield due to this LET dependent process is much larger than the normal levels. A full mechanistic description of this effect is still missing.

Another limitation of the present results is arising from the non-negligible difference in sensitivity found in chronic hypoxia (the condition most relevant in clinical cases) as compared to acute hypoxia (the condition verified in the present experiments). We recently initiated the analysis of the effect of ion beams in chronic hypoxia^49^, and once extensive measurements will be accomplished also for this condition, they could be transferred in the same way to the present approach. Finally the use of CHO cells is motivated, beside their simplicity and standardized handling protocols, by their α/β ratio, similar to a sensitive human tumour tissue. Moreover in a previous work^8^, OER measurements for Chinese hamster cells (V79), were similar to human salivary gland (HSG) cells. However the validation of the present model with selected data points collected for human cells will provide an important outlook of the present work, before its clinical implementation.

The present approach exploits the use of charged particles and compared to photon dose painting presents several advantages. Increasingly important research has been carried in this field^22^,^30^,^46^, where in principle a similar compensation of the hypoxia induced radioresistance is performed only by means of increasing the radiation dose, while having the same limitations in terms of accuracy of the PET imaging. The latter method, however, appears to be less convenient for two aspects. Firstly a general increase of dose in the entrance channel with photons has a larger impact on the normal tissue than a similar increase in particle therapy, where normally the number of irradiation ports are much lower. Moreover the lack of LET as an additional relevant parameter, imposes an absolute larger amount of dose to be boosted in the hypoxic regions to overcome OER.

### Conclusions and outlook

The kill-painting approach is a biologically driven inverse planning technique with a new optimization quantity. The biological isoffective dose is optimized in the local tumour microenvironment. This allows exploiting simultaneously the potential advantages of functional imaging and the flexibility of actively scanned particle therapy.

Kill-painting allows optimization of treatment plans in the presence of any type of intra-tumour heterogeneity. Heterogeneity is emerging as an important prognostic factor, and targeting multiple tumour compartments is a possible solution to fight radioresistance and achieve sustainable responses^50^. The input in this case should be an intra-tumour map from a specific functional imaging tool, and a model analogous to the present one for the corresponding sub-volume radiosensitivity, including its LET dependence. Examples are stem cells niches^14^, which could be identified by imaging their related marker CD133 by ^64^Cu-ATSM tracer (copper- diacetyl-methylthiosemicarbazone)^51^, or even with near-infrared fluorescence molecular tomography^52^, and regions of increased repair activity through γH2AX labeling^53^,^54^.

## Materials and Methods

### Cell culture

At GSI, Chinese Hamster Ovary cells (CHO) were obtained from the American tissue culture collection. At NIRS, CHO cells were provided by the RIKEN BRC. They were grown in Ham´s F12 medium (SIGMA) supplemented with 10% fetal bovine serum and antibiotics (100 U/ml penicillin and 100 μg/ml streptomycin all Biochrom AG, Berlin, Germany) under humidified air with 5% carbon dioxide at 37 ^o^C.

For the GSI experiments, cells were seeded in a ring consisting of polyvinyl-chloride and with an internal diameter of 24 mm and a width of 3 mm at a concentration of 5 × 10^4^ one day before the irradiation. Both sides of the sample ring were covered with a gas permeable foil of 25 μm thickness (BioFolie25, *in vitro* Systems and Services, Göttingen, Germany). One layer of foil corresponds to a water-equivalent thickness of 47 μm.

For the NIRS experiments cells were harvested with 0.05% Trypsin-EDTA and seeded in 3.8 cm-diameter glass dishes at a concentration of 2 × 10^5^ one day before the irradiation.

### Colony forming assay

After irradiation cells were rinsed with PBS. For harvesting, cells were treated with trypsin, 5 minutes at 37 ^o^C and then re-suspended in 1 ml of fresh medium. Cell number was calculated with a Coulter counter analyser (Beckman Coulter). Approximately 100 surviving cells were re-seeded per 6 cm plastic dishes in 5 ml of fresh medium. For each irradiated point of each survival curve, 3 different dishes were prepared.

After re-seeding, cells were incubated for 7 days at GSI and 12 days at NIRS, according to the locally established protocols, enough for the surviving cells to proliferate over 50 clones/colony.

The resulting colonies were fixed with 10% formalin in PBS for 10 minutes, and stained with 1% methylene blue in water. Colonies consisting of more than 50 cells were counted as survivors.

Every survival measurement was repeated in 3 independent experiments (biological replicates), where for each biological replicate the colonies counting was performed from 3 separate samples (technical replicates). For every measurement at given oxygen concentration a simultaneous irradiation in fully oxic conditions is performed in order to have a contemporary ‘oxic control’.

### Hypoxic chambers

#### a) GSI chambers

For the irradiation under hypoxic conditions, at GSI special designed and patented exposure chambers have been used, which allow irradiating of cell cultures with X-rays or ions under defined oxygenation conditions^55^.

The chamber is cut from a single piece of polyetheretherketone (PEEK). The top cover is transparent polymethylmethacrylate (PMMA) to allow position control of the sample. The front wall is used as irradiation window and has a thickness of 1mm, 1.23 mm water equivalent. A system of hose couplings allows to gas the chamber and to keep it gas-tight afterwards for irradiation. Since PEEK and PMMA are known to contain dissolved oxygen, the possible release of oxygen inside the chambers after irradiation was tested by measuring the actual concentration with a fiber-optic oxygen meter, revealing no measurable effect.

Two different devices have been used. Single sample irradiation chamber (SHC) or multiple samples hypoxic chamber (MHC, 3 samples at the same time).

#### b) NIRS chambers

At NIRS a multi-sample hypoxic chamber was used. The chamber is made of steel and has 8 different irradiation windows in 2 rows (X-rays hypoxic chamber, horizontal irradiation^56^) or 7 in one line (ion hypoxic chamber^57^). The Petri dishes used for the ion irradiation have a small pocket on one side to contain the medium during the irradiation (see Fig. 5d). For the ion irradiation, samples are in vertical position. This peculiarity gives the possibility to keep high humidity atmosphere during irradiation. Cells were not seeded in the area where the pocket is placed, to avoid beam degradation and different LET in the same sample.

### Monoenergetic Irradiation

Every measurement was performed at a given dose averaged LET, which is defined as the summation of the LET of all the individual particles composing the beam weighted for their relative doses




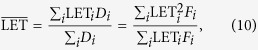




with LET_i_, *D*_*i*_ and *F*_*i*_, denoting LET value, dose and fluence, respectively, of the individual beam component *i*.

#### a) NIRS-HIMAC

To obtain the required dose averaged LET, a passive beam has been used at HIMAC.

Carbon ions having a 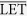 from 30 to 300 keV/μm were provided by a primary energy beam of 290 MeV/nucleon.

Silicon ions having a 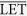 of 400 keV/μm were provided by a primary energy beam of 490 MeV/nucleon.

X-ray irradiation was produced by a generator (SHIMADZU, PANTAC HF-320S) operated at 200 kVp and 20 mA^58^.

#### b) GSI-SIS and HIT

To obtain a sufficiently high LET, the chambers were irradiated with an extended 3D-plan covering 1cm in depth, using a bolus of 30 mm and 5 ion energies. All the samples were irradiated with an actively scanned beam.

Nitrogen and Carbon ions experiments were performed at SIS in GSI, while the Oxygen beam experiment was performed at HIT, Heidelberg. Nitrogen ions had a 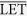 of 160 keV/μm and Oxygen ions had a 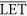 of 140 keV/μm.

X-ray irradiation was carried out with a Isovolt DS1 X-ray machine (Seifert, Ahrensberg, Germany) operated at 250 kVp and 16 mA with 7 mm beryllium, 1 mm aluminum and 1 mm copper filtering and a dose rate of 2 Gy/min.

### Extended target irradiation

For the extended target irradiation, used to simulate therapy conditions with a complex tumour with variable oxygen concentration, a phantom was assembled by combining tissue culture flasks (TCF) and hypoxic chambers (HC) in order to increase the number of measurement points along the entrance channel and the target region. The full length was 18 cm at NIRS and 16 cm at GSI in water equivalent (weq) along the beam directions, while the target was placed in the middle with a lateral extension of 4 cm × 4 cm and a depth of 6 cm (weq).

In the NIRS irradiation 2 opposite fields passively shaped with a 6cm spread out Bragg peak (SOBP) were superimposed, rotating the sample by 180 degrees between the two irradiation shots. The delivered dose in this case was higher than it was planned, due to a dosimetry problem at the accelerator, but the actual dose was recalculated with respect to the oxic control data, and then the full plan was recomputed with this dose (9.5 Gy(RBE)) for the differently oxygenated regions.

At GSI the irradiations were performed in Cave M, with full active scanning capabilities. The samples were placed on a support tilted by 2.203 degrees in order to compensate the fixed inclination of the beam pipe and obtain a real 180 degrees irradiation, which emphasize the efficiency of the kill-painting method. The beam energy spacing was chosen in general for depth steps of 3 mm, while at the boundaries between different sensitivity areas, the energy slices were selected in order to avoid occurrence of the peak close to the boundary and consequent fluctuations in the biological effect. A triple chamber hosting three samples at a given concentration and different depth positions was used. In order to minimize range uncertainties and to reduce the overall size of the phantom, the chambers with different concentration were not piled one after the other, but irradiated on their turn with the same plan, each one placed at the corresponding depth position in water equivalent (see Fig. 5). This was realized in 3 irradiations for each configuration, grouped into 2 beam times performed at GSI.

### Treatment Planning

#### a) TRiP98

All treatment plans were done using an extended version of the TRiP98 treatment planning system. TRiP98 (Treatment planning for particles) is the first treatment planning system dedicated to active scanned carbon ion therapy. It was in use for all the irradiations of the GSI pilot project. Nowadays it serves as a research tool in several facilities as well as a reference for commercial planning systems dedicated to active scanned particle therapy. Among the main features of TRiP98 is the possibility to consider the contribution of all the particles composing the mixed irradiation field.

The general structure of the code, including the present implementation, is available in the SI, while further details are available in refs 33,42, 43, 44,59,60

#### b) Kill-painting implementation

The main task of the optimization module is the minimization of an objective function 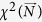, which is dependent on the particle numbers within each spot of the raster scanning (components of the vector 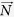) and is including the deviations, at any voxel *i*, of the prescribed dose 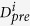 to the actual 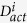 at any updating iteration of the vector 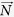.




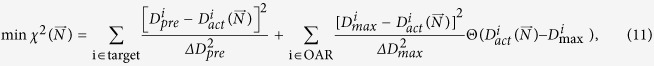




where the second sum is running over the organ-at-risks (OAR) voxels and is modulated by the Heavyside function *Θ*, evaluating to 1 when a maximum allowed dose 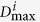 is exceeded, and otherwise to 0. 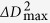 and 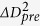 are adjustable weights for the two contributions, allowing to choose between OAR sparing and target dose conformity.


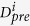
 is normally a biologically effective (RBE-weighted) dose generated by the contributions of all the beam components to a given voxel^59^




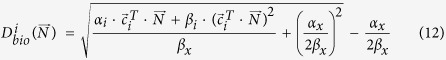




where the 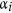 ,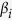 are the ion parameters after dose averaging in the mixed radiation field resulting on the voxel i, and the 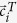 are terms representing the contributions of all the raster spots to the voxel (*60)*.

In order to consider an “isoeffective dose accounting for hypoxia”, we then modified the values of these parameters at any voxel, accounting for the local OER value, depending on a local constant quantity 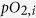, and another quantity (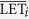) which is updated in the course of the optimization,




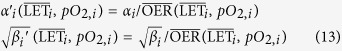




The latter decomposition is possible thanks to the dose modifying nature of the 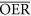 quantity.

Since the algorithms of biological dose optimization are mainly gradient based, it is necessary also to modify the gradients of the biological effect with respect to the particle numbers. Thus, similarly, a modification of the gradients driving the minimization of the objective function is necessary,




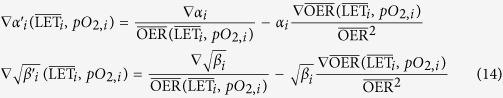




assuring that the particle numbers and the LET distribution update is driven by the OER effect.

## Additional Information

**How to cite this article**: Tinganelli, W. *et al*. Kill-painting of hypoxic tumours in charged particle therapy. *Sci. Rep*. **5**, 17016; doi: 10.1038/srep17016 (2015).

## Supplementary Material

Supplementary Information
