# Supplementary material for: Kill-painting of hypoxic tumours in charged particle therapy
Source: Sci Rep. 2015 Nov 24;5:17016. doi: 10.1038/srep17016 (PMC4657060; doi:10.1038/srep17016)
Supplement: Supplementary Information [file srep17016-s1.pdf]

Supplementary Information:

Kill-painting of hypoxic tumors in charged particle therapy

Walter Tinganelli[1,2,], Marco Durante[1,3]*, Ryoichi Hirayama[2], Michael Krämer[1], Andreas Maier[1], Wilma Kraft-Weyrather[1], Yoshiya Furusawa[2], Thomas Friedrich[1], Emanuele Scifoni[1]

[1] Biophysics Department, GSI Helmholtzzentrum für Schwerionenforschung, 64291 Darmstadt, Germany.

[2] Research Center for Charged Particle Therapy and International Open Laboratory, National Institute of Radiological Sciences, 263-8555 Chiba, Japan.

[3] Technical University Darmstadt, 64283 Darmstadt, Germany.

Supplementary Materials and Methods

Gas modalities

For the GSI and HIT experiments, gas flow was measured with a thermal mass flow meter calibrated for nitrogen (red-y, flow technology by Vögtlin). The standard experimental protocol was 2 h gassing with 200 ml/min. Standard-gas mixtures used were 95% N_2; 5% CO_2 (anoxia) and 94.5% N_2; 5% CO_2; 0.5% O_2 (hypoxia). To determine the required time and the gas flow to reach the planned oxygen state in the medium, the oxygen pressure was measured using a Needle-Type housing optical O2 micro sensor (Pre-Sens, Regensburg, Germany).

At NIRS, the chambers were flushed with a gas mixture consisting of 1000 ml/min of air, pure carbon dioxide and nitrogen for 1 hour immediately before irradiation. These gasses were passed through a bubbling bottle to maintain high-humidity levels before flowing into the chambers.

Different gas mixture combinations were used:

- 0% oxygen concentration (anoxia) [1]
- 0.15%, 0.5%, 2% oxygen concentration (hypoxia)

TRiP98 Structure

An overview of the entire structure of the TRiP98 treatment planning code, including the present implementation, is shown on figure S1. The input in a specific planning task is mainly composed of data on the patient, conventionally provided by computer tomography (CT) scans and contours, providing a morphological map as usual in radiotherapy. In the present extension we allow also the transmission of some functional information, i.e., a pO_2 map, on a voxel by voxel basis, which can be obtained for example by functional imaging scans. Besides patient information, part of the input is also a description of the specific

irradiation setup. For this aim the code is rather flexible and usable in different facilities, allowing to account for different beams, both in passive and active modality.

The modular structure is conceived in order to allow importing of tables describing the monoenergetic, single ion components of physical and biological effects, which can always be improved and updated without modification of the code itself. For example, among these tables, which are constantly tuned with measurements, there are for the physics the ion fragmentation spectra, or for the radiobiology the intrinsic RBE_α tables, which are normally produced with the LEM model. The OER tables described here represent a new type of radiobiological information. The combination of all these informations for a realistic macroscopic beam is then processed in the main core of the code, which is based on two main sections: the physical beam model and the biological effect calculation. These tools process the input datasets with specific algorithms, e.g. for mixing the biological effect of all the beam components. The optimization process is then updating the field, i.e. the vector of all the particle numbers for every raster position and energy, according to equation (12) in an iterative way for an inverse planning task and is finally returning the optimized scan parameters, as well as the 3D planning profiles (dose, LET, survival, RBE etc.).

Further details on the code structure are available in Refs[2-7].

Oxygen Enhancement Ratio (OER) calculation

We consider the OER as the quantity defined in eq.1, despite some authors adopting a different naming for the same quantity, i.e. 'hypoxia reduction factor' (30), leaving instead the OER term to its complementary value, obtained by the ratio of doses respectively at the given concentration and in fully anoxic condition.

All the dose-response curves were fitted by the linear quadratic (LQ) equation (eq.3). Data from three independent experiments were pooled in a single curve linear quadratic fitting, returning the two parameters α and β with their corresponding standard errors (σ) of the fit. The OER(S) for a generic survival level S was then calculated, according to eq. 4, and the value of OER(10%), was taken as a good approximation of a constant dose modifying factor [7]. For the highest LET values, where a LQ fit was found inconsistent, a purely linear response was adopted.

The corresponding error on the OER(10%) was then calculated from the error propagation, i.e. analytically deriving the full function of equation 4, for S=10%, with respect to the two α parameters, neglecting the contribution on the error of the β parameters, as

$$\sigma_{\mathrm{OER}} = \sqrt{\left(\frac{\partial \mathrm{OER}}{\partial \alpha_{\mathrm{ox}}}\right)^2 \sigma^2_{\alpha_{ox}} + \left(\frac{\partial \mathrm{OER}}{\partial \alpha(pO_2)}\right)^2 \sigma^2_{\alpha(pO_2)}}, \tag{S.1}$$

The overall behaviour of the cells was found fairly regular and reproducible, with remarkably high correlation values. At the highest dose point in the hypoxic curve, a consistent deviation from the curve was, however, often observed. These points, probably originating from a slight concentration gradient occurring in the chambers, inducing a finite size distribution of sensitivities, were thence excluded from the fitting, after observing that their inclusion would produce a fit with unrealistic curve bending (positive β values) and with a much lower correlation coefficient

In Table S1 all the OER data extracted by the present measurements are collected, corresponding to the experimental points in figure 3. In order to integrate our original data with a number of previous measurements performed in full anoxia at GSI and HIT [8], for

consistency the same fitting procedure mentioned above was applied also to the raw data of these experiments, obtaining OER values slightly different (within a 5% deviation) to those reported there. For the oxygen beam, at HIT, only one experiment was done. For this single experiment then the OER was calculated from the 2 single survival curves (oxic and anoxic) and the error was derived from the technical replicates.

Additional Controls

For the extended target irradiations a complete normoxic control was performed (see fig. S2a). This serves to test the irradiation fields and the treatment plan in normal conditions, e.g. to exclude possible effects of delayed irradiation between the two fields, and also to indicate the resolution of the hypoxic chambers, where local gradients of concentrations are unavoidable and induce an appreciable spread in the measurements.

In a further experiment (see fig. S2b) we irradiated with an OER optimized plan normoxic cells placed in the same position where the anoxic ones were situated, i.e. at the center of the target (exactly at 77 mm depth), in order to observe the effect of the adapted plan on a normoxic tissue and to disentangle the contributions of RBE and OER, dose and LET on the overall biological effect. The latter experiment, performed in 3 biological replicates, returned for these cells a survival level of $(6.2\pm1.3)\times10^{-3}$, which was in agreement with our computed expected survival, 5×10^{-3} . This survival value is corresponding to an increase in RBE weighted dose up to 11.3 Gy(RBE) from the originally planned 6.5 Gy(RBE), i.e. of a factor 1.74. This factor is much lower than the factor 2.5 which would be required in a normal irradiation (see, e.g., Fig.3), considering that at the center of the target in a normoxic plan $\overline{\mathrm{LET}}$=55 keV/µm. This means that the achieved compensation of the anoxic cells resistance is indeed not simply a result of a dose increase, but it involves at the same time a decrease of the OER effect.

Moreover, in order to test the consistency of measurements performed by combining different supports for the cell irradiation, as we did mixing tissue culture flasks (TCF) and hypoxic chambers (HC) in our phantom, we measured and compared X-ray survival curves for both samples. Measurements were performed directly after the last extended target experiment and were compared to other independent experiments on TCF in order to assess the relevance of the deviations, as compared to the intrinsic variability of the cell samples. The results (see fig. S3) show a negligible effect, which allows to safely consider the different samples as an identical biological target.

OER optimization analysis

In figure S4 we show an analysis of the pencil beam fluences, as computed for the two plans for a single raster spot in the center of every energy slice. The contribution of selected monoenergetic pencil beams to the overall dose are shown. It is evident that while in a normoxic plan the highest particle numbers are in the respective distal energy slices, in the OER-optimized case they are concentrated in the most hypoxic region, i.e., in the center of the target. The resulting redistribution implies a $\overline{\mathrm{LET}}$ increase in the target center from 55 up to 75 keV/µm, according to our calculations. On the other hand, in order to induce without any dose boost the observed strong decrease of the OER, the $\overline{\mathrm{LET}}$ should be in the order of 200 keV//µm, a condition which is hardly realized in a carbon ion plan. Therefore the resulting survival, achieved by the kill painting, is really due to a combination of dose and LET redistribution. Moreover, the LET increase has the additional effect of increasing the RBE,

besides reducing the OER. We found, however, that RBE in the target center was almost constant between the two plans (1.41 versus 1.42). The reason for this is that the simultaneous increase of the dose reduces the RBE. The two effects (LET and dose increase) are then acting in the opposite way and, in this case, compensating the RBE change, while the OER decrease is responsible for the enhanced sensitization. This is a further demonstration that in a general case it is mandatory to consider at the same time, dose, LET, RBE and OER for a full biological based optimization, rather than imposing a single prescribed quantity.

Supplementary Figures and Tables

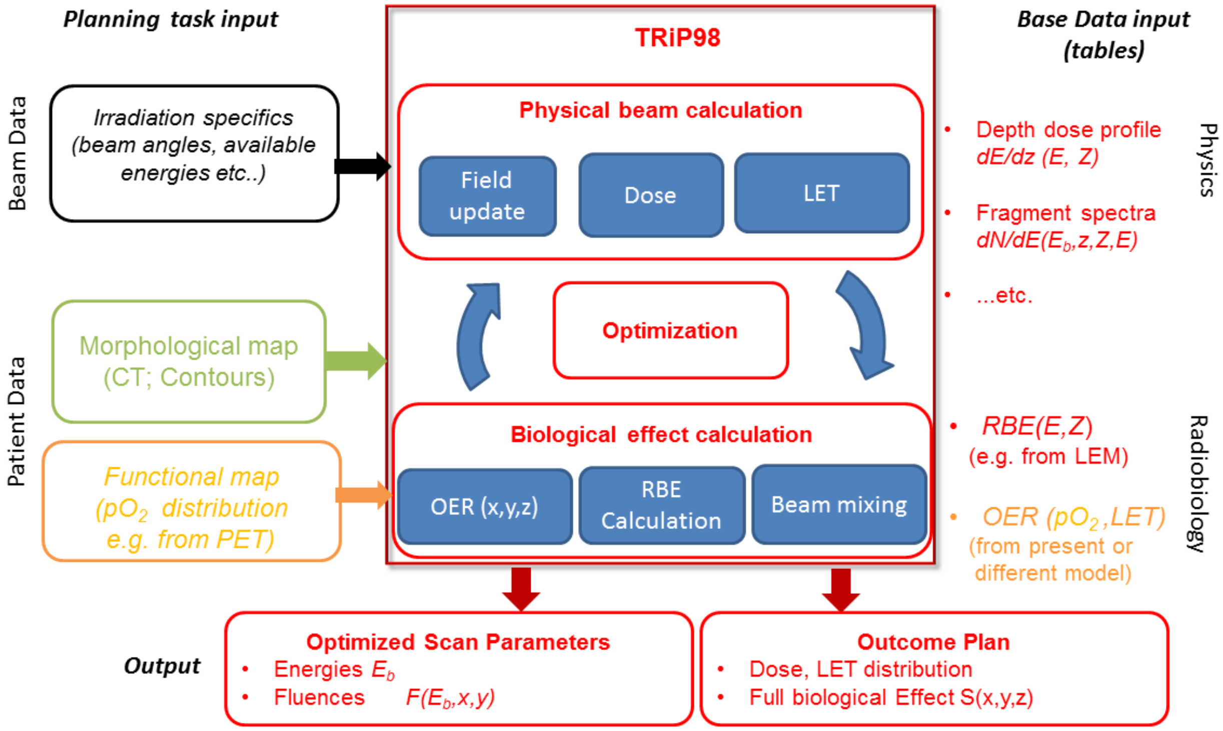

Fig. S1: TRiP98-OER flowchart. Scheme of the TRiP98 code, including the present implementation, allowing the input and processing of oxygenation information.

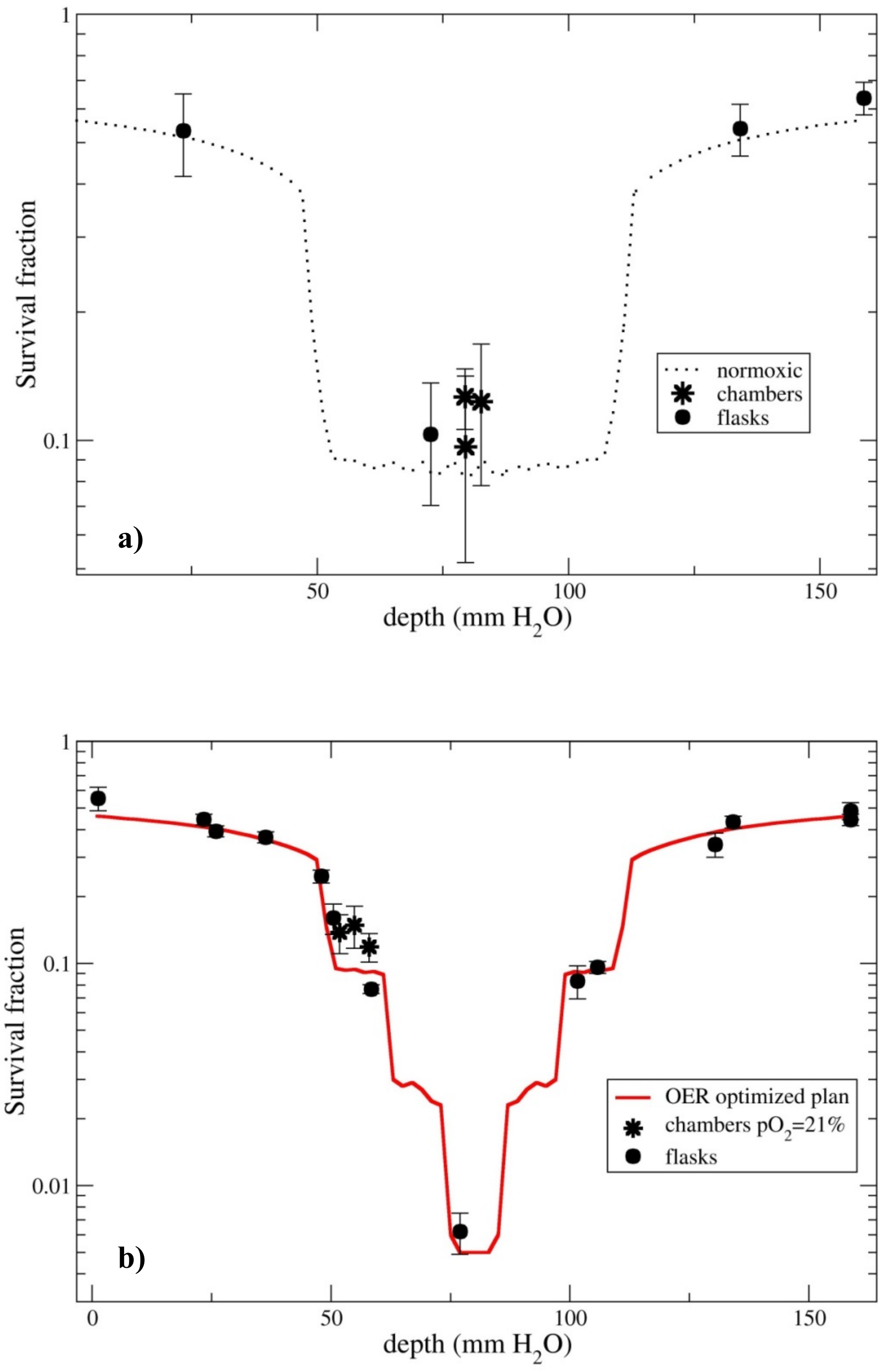

Fig. S2 Normoxic controls in the extended irradiated geometry. Survival results from 2 opposed fields irradiation in fully oxic conditions, measured by the hypoxic chambers (pO_2=21%) and tissue culture flasks. a) Normoxic plan irradiation, with planned RBE-weighted dose in the target 6.5 Gy (RBE). b) OER optimized plan, as in Fig. 7, applied to normoxic cells. The point in the center (77 mm H2O) is obtained irradiating normoxic cells placed in the region considered for anoxic cells in the optimization. The other points are the same as in Fig. 7.

Facility	Ion species	LET (keV/µm)	pO_2	N. exp	α_{ox} (Gy^{-1})	β_{ox} (Gy^{-2})	$\alpha(pO_2)$ (Gy^{-1})	$\beta(pO_2)$ (Gy^{-2})	OER(10%)
**NIRS**	X-rays	9.4	0%	3	0.43[0.12]	0.095[0.020]	0.14[0.02]	0.013[0.001]	2.83[0.37]
**GSI***	X-rays	9.4	0%	3	0.16[0.01]	0.020[0.001]	0.09[0.01]	0.003[0.001]	2.40[0.10]
**NIRS**	X-rays	9.4	0.5%	3	0.43[0.12]	0.095[0.020]	0.36[0.07]	0.016[0.006]	1.62[0.30]
**GSI***	X-rays	9.4	0.5%	3	0.16[0.01]	0.020[0.001]	0.14[0.02]	0.008[0.001]	1.50[0.10]
**NIRS**	Si	308	2%	3	0.88[0.01]	-	0.79[0.01]	-	1.07[0.07]
**NIRS**	Si	283	0%	3	1.02[0.02]	-	0.75[0.01]	-	1.35[0.12]
**NIRS**	Si	304	0.5%	3	0.87[0.05]	-	0.90[0.08]	-	1.08[0.11]
**HIT***	O	140	0%	1	0.71[0.03]	-	0.50[0.12]	-	1.44[0.20]
**GSI***	N	160	0%	1	0.78[0.02]	-	0.59[0.06]	-	1.30[0.04]
**NIRS**	C	68.1	2%	3	1.09[0.02]	-	0.91[0.02]	-	1.20[0.13]
**NIRS**	C	51.2	2%	3	0.54[0.03]	0.082[0.008]	0.63[0.05]	0.023[0.010]	1.12[0.09]
**NIRS**	C	29.8	2%	3	0.43[0.04]	0.057[0.008]	0.38[0.04]	0.038[0.008]	1.18[0.14]
**NIRS**	C	317	0%	3	1.20[0.07]	0.022[0.016]	0.95[0.04]	0.011[0.007]	1.28[0.09]
**NIRS**	C	141	0%	3	1.14[0.09]	0.046[0.020]	0.70[0.06]	0.040[0.012]	1.49[0.19]
**NIRS**	C	51.1	0%	3	0.75[0.06]	0.049[0.014]	0.29[0.01]	0.010[0.001]	2.46[0.23]
**GSI***	C	150	0%	2	0.79[0.04]	-	0.61[0.02]	-	1.33[0.05]
**GSI***	C	100	0%	3	0.81[0.01]	-	0.41[0.02]	-	1.81[0.12]
**NIRS**	C	124	0.5%	3	1.14[0.05]	0.038[0.011]	1.03[0.08]	0.007[0.018]	1.16[0.09]
**NIRS**	C	51.5	0.5%	3	0.62[0.03]	0.086[0.006]	0.57[0.07]	0.009[0.012]	1.40[0.15]
**GSI***	C	100	0.5%	3	0.81[0.01]	-	0.64[0.04]	-	1.29[0.08]
**NIRS**	C	124	0.15%	2	1.14[0.05]	0.038[0.011]	0.88[0.08]	0.007[0.016]	1.34[0.12]
**NIRS**	C	68.0	0.15%	3	0.84[0.07]	0.069[0.016]	0.51[0.04]	0.027[0.007]	1.64[0.18]
**NIRS**	C	29.8	0.15%	3	0.37[0.03]	0057[0.007]	0.31[0.03]	0.001[0.003]	1.90[0.16]

Table S1: Measured OER data. List of performed experiments and extracted experimental OER values from the plots like in figure 1 (standard error in brackets) and corresponding information about the irradiation conditions. Fitted LQ parameters are also listed for each condition ($\alpha(pO_2)$, $\beta(pO_2)$) and corresponding oxic control (α_{ox}, β_{ox}). * Data from GSI and HIT are reevaluation of experiments published on Ref. [8]. All data from NIRS are from original measurements.

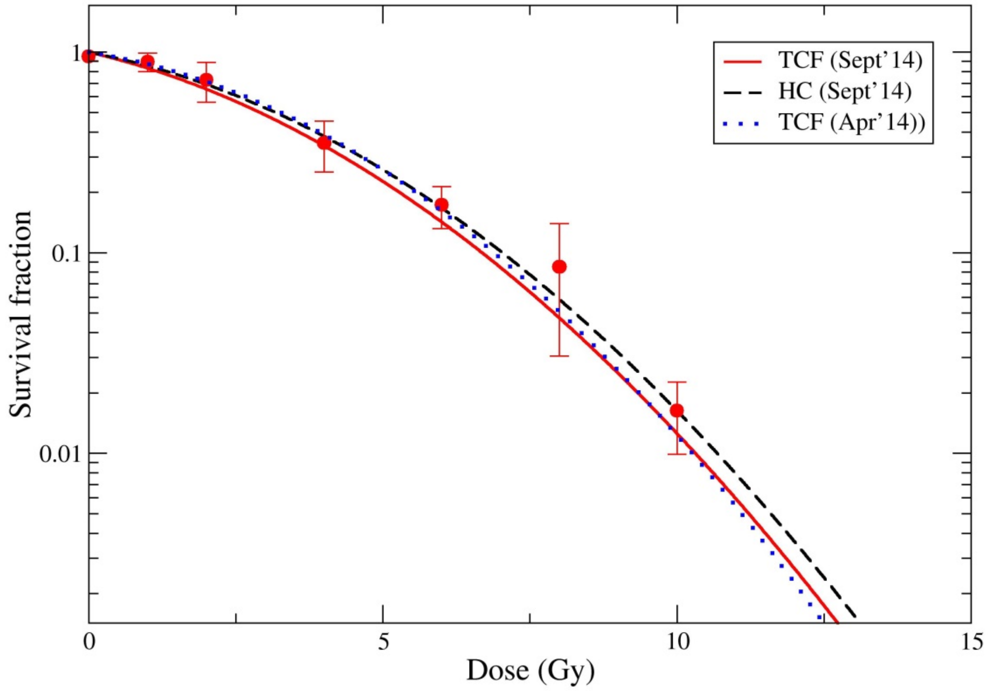

Fig. S3: Impact of cell support on radiosensitivity. Comparison of the radiosensitivity of CHO cells, when grown on the different devices used for the verification experiments in the present work (tissue culture flasks (TCF) and hypoxic chambers (HC)), as measured a few days after the last beam-time (September 2014). The reference curve (April 2014) represents an independent measurement on TCF, performed after the first beam time, to show the intrinsic variability. All curves are obtained from three independent experiments. Only for the first curve, also the measured points are shown (circles, average and standard deviation).

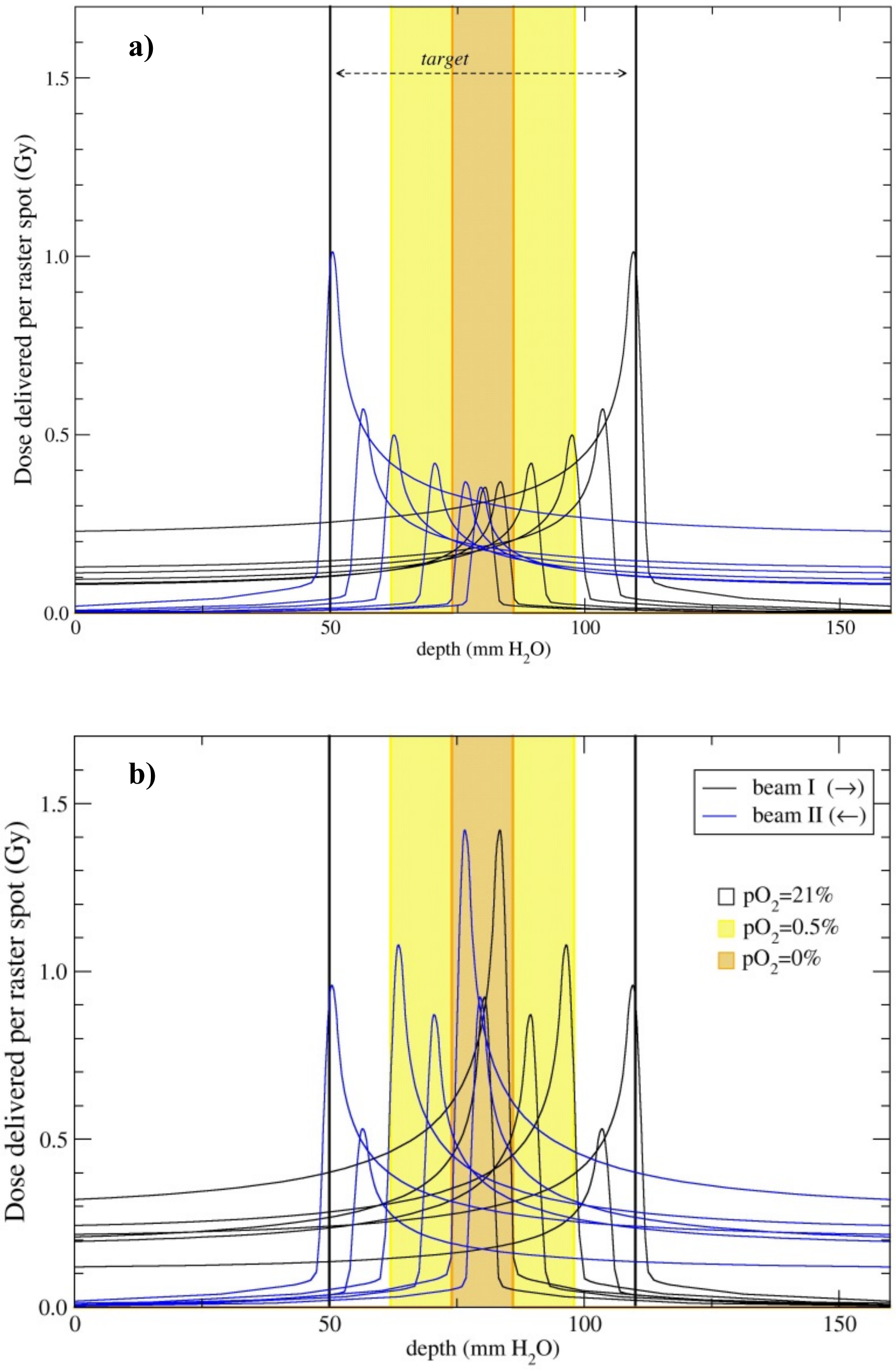

Fig. S4: OER optimization details. Redistribution of the particle fluences for the single monoenergetic pencil beams in the OER optimization, for a central spot of the raster scanning system. Panel (a) normoxic plan, (b) OER-optimized. For the sake of clarity, contributions to the dose distribution only from a few selected energy slices are shown.

1 Hirayama, R. *et al.* Radioprotection by DMSO in nitrogen-saturated mammalian cells exposed to helium ion beams. *Rad. Chem. Phys.* 78, 1175-1178, (2009).

2 Krämer, M. *et al.* Treatment planning for heavy-ion radiotherapy: physical beam model and dose optimization. *Phys. Med. Biol.* 45, 3299-3317, (2000).

3 Krämer, M. & Scholz, M. Treatment planning for heavy-ion radiotherapy: calculation and optimization of biologically effective dose. *Phys. Med. Biol.* 45, 3319-3330, (2000).
4 Krämer, M. & Scholz, M. Rapid calculation of biological effects in ion radiotherapy. *Phys. Med. Biol.* 51, 1959-1970, (2006).
5 Krämer, M., Scifoni, E., Schmitz, F., Sokol, O. & Durante, M. Overview of recent advances in treatment planning for ion beam radiotherapy. *Eur. Phys. J. D* 68, 306, (2014).
6 Krämer, M., Scifoni, E., Wälzlein, C. & Durante, M. Ion beams in radiotherapy - from tracks to treatment planning. *J. Phys. Conf. Ser.* 373, 012017, (2012).
7 Scifoni, E. *et al.* Including oxygen enhancement ratio in ion beam treatment planning: model implementation and experimental verification. *Phys. Med. Biol.* 58, 3871-3895, (2013).
8 Tinganelli, W. *et al.* Influence of acute hypoxia and radiation quality on cell survival. *J. Radiat. Res.* 54, i23-i30, (2013).
